# Empathic Psychology: A Code of Risk Prevention and Control for Behavior Guidance in the Multicultural Context

**DOI:** 10.3389/fpsyg.2021.781710

**Published:** 2021-11-24

**Authors:** Kui Yi, Yi Li, Huaxin Peng, Xingrong Wang, Rungting Tu

**Affiliations:** ^1^College of Economics and Management, East China Jiaotong University, Nanchang, China; ^2^Media and Communication School, Shenzhen University, Shenzhen, China; ^3^College of Management, Shenzhen University, Shenzhen, China

**Keywords:** crisis events, empathy communication, public empathy, risk prevention and control, multicultural context

## Abstract

This study aims to uncover the relationship among multicultural differences, empathy, and the behaviors of risk prevention and control in the context of crisis events by using a sample of 300 individuals in 10 different multicultural countries. A theoretical logic model was applied to empirical analysis, and the results indicated that cultural differences positively influenced the behavior of empathy communication and risk prevention and control. Further analyses revealed that real-time monitoring of changes in empathy could provide better options of measures for local risk prevention and control when the same crisis event occurred in a multicultural context. With user-generated content (UGC) emerging in the web 2.0 era, this paper proposed a more profound empathy code regarding the periodicity of risk prevention and control. This paper expects to contribute to the circumvention of cognitive errors caused by cultural differences, and to further provide effective conduction for individuals' risk prevention and control behaviors.

## Introduction

As social crises occur frequently, both industry and academia have paid much attention to the social damage caused by both insufficient and excessive risk prevention and control. In industry practice, practitioners have attempted to seek ways to reduce the overall damage caused by crisis events and to coordinate losses in various aspects. Meanwhile, exploring the reasonable scope of risk prevention and control has become hot topics in academia. With the expansion of economic globalization, cultural exchanges among countries have become more frequent, while public safety emergencies are also on the rise. Advances in the Internet have accelerated the emergence of the user-generated content (UGC) model, which has led to a dramatic change in the way the public disseminates information. Public safety has a profound impact on the individuals with different concepts of risk prevention and control in the multicultural context. These impacts generally stem from the failure to overcome the negative influences of a crisis, or from the huge changes in lifestyle that cause individuals to be uncomfortable with the new social lifestyle. As far as the relevant authorities are concerned, neither of these situations are ideal, but both are often equally inevitable. Therefore, how to properly handle risk prevention and control in a normal lifestyle has become an important public management issue.

The appearance of behavior is usually driven psychologically. Nowadays, the transmission of information is more convenient and faster. Once a crisis event occurs, it will inevitably affect the psychological attitudes of the public, which in turn affects behaviors. Public psychology is at the core of explaining the phenomenon of psychological resonance in crisis events, which has been well-studied in the medical psychology I. The most cited concept is empathy of Rogers (1958), which mainly focuses on the process of individual perception while considering the resulting emotional change. Recently, the concept of empathy has become common in the fields of management, sociology, communication, and education (Tansey and Burke, 2013).

We attempt applied the psychological concept of empathy to explore the current hot issues in public risk prevention and control from the perspective of empathy, and explores the general logic of risk prevention and control behaviors occurring in the context of UGC. Specifically, we summarized these hot issues are into four sub-questions: the first is whether different cultural differences lead to different risk prevention and control behaviors of the public; the second is whether empathy presents different characteristics in different cultural contexts; the third is whether empathy exerts a direct influence on the occurrence of risk prevention and control behaviors; and the fourth is whether the process of public risk prevention and control changes in the context of UGC and the law of occurrence. The solutions to the four research questions will systematically analyze the psychological black box of behavior guidance on risk prevention and control under the background of cultural differences and provide theoretical enlightenment for countries and regions to respond to crisis events.

## Literature Review and Hypotheses Development

Previous studies on risk prevention and control under the background of cultural differences, not only revealed the laws of multicultural differences and social conflicts, but also showed the significance and application of empathy communication in crisis prevention and control, reflecting the proactive research prospects. Our study aims to enrich the literature of active risk prevention and control behaviors in the multicultural context and further solidate the research basis.

### Multicultural Differences and the Occurrence of Social Conflict

Diversity is an important trend of contemporary cultural development, and the conflicts caused by its different manifestations are the key factors in the occurrence and evolution of crisis events. The concept of cultural difference was first proposed by Hofstede (1980), who categorized culture value into four dimensions “power distance index,” “uncertainty avoidance index,” “individualism-collectivism,” and “masculinity-femininity.” The concept has been widely applied to explain the cultural heterogeneity between different cultures (Li and Katsumata, 2020; Liu et al., 2021). The view that heterogeneity of cultural value triggers secondary conflicts in public safety emergencies has been widely recognized by academics in studies related to cross-cultural conflicts (Nielsen and Lockwood, 2016).

However, with the increasing findlings of internal and external factors, scholars believe that the factors affecting culture are not limited to a single dimension, but the results of the interaction of multiple dimensions, thus giving rise to the concept of multiculturalism. From the 1970s to the present, the process of globalization has been intensifying; the differences between cultures have become more pronounced; the resulting conflicts have become more acute; and multiculturalism has become the focus of continuous research in the European and American academia. However, the concept of multiculturalism still has no clear definition. It has different uses and connotations in different fields. This concept shows its inherent educational thought through historical and literary critical theory, which is a mixture of political attitude and ideology (Maddux et al., 2021). Specifically, the conflicts caused by multicultural differences are high-dimensional manifestations of cross-cultural differences and conflicts, and multicultural differences involve more complicated influencing factors than cross-cultural differences. Therefore, the study of cultural differences and conflicts in multicultural countries should be based on the study of cross-cultural differences and conflicts, as well as the heterogeneity of cultural values, in order to deeply explore the roots of cultural differences and conflicts in these countries that are affected by multidimensional factors. Based on the cultural dimension theory of Hofstede (1980), scholars such as Kogut and Singh (1988) proposed the concept of cultural distance. They believe that relevant research should follow the conceptual framework of cultural distance index analysis and transform the conceptual understanding of national borders into cultural borders and attribute cross-cultural conflicts to the heterogeneity of cultural values at the national level (Kogut and Singh, 1988; Beugelsdijk et al., 2017). Previous studies analyze cross-cultural conflicts through the cultural value perspective (Lu, 2015).

To sum up, the main influencing factor of cultural value dimensions on cross-cultural conflicts is essentially the heterogeneous manifestation of the existence of cultural values. However, the cultural composition in a multicultural context is more complex. It is affected by factors such as the differences in region, culture, nationality, and domestic education levels, as well as external factors such as international cultural differences, world ethnic cultural differences, and international differences. Existing studies suggest that this framework can serve as a guide to the analysis of cultural differences and conflicts in multicultural countries.

Cultural distance is a widely used measurement constructed in international business, and this distance measurement is particularly important in a global multicultural context (Sekiguchi, 2006; Li and Katsumata, 2020; Li et al., 2021). How to measure the differences more accurately between cultures is the key to resolve crisis events in multiculturalism. The perceived cultural differences in multiculturalism are related to the inclusiveness of multiculturalism itself (Alexandra et al., 2020). Specifically, actual conflicts are not caused by multiculturalism, while the social crisis caused by cross-cultural differences originate from the information asymmetry in multiculturalism (Xie, 2021). Accordingly, cultural differences have both common features and individual differences within and across countries. Previous literature has uncovered that crisis events caused by cultural differences can be resolved through predetermined solutions, and cross-cultural differences has strong impacts on individual attention, perception, and psychology (Gutchess et al., 2021) and even play a certain role in guiding individual response mechanisms in the face of crisis events. It is also worth studying which factors in unexpected crisis events affect individuals in a multicultural context and how these factors originate and arise. Exploring the interaction between individuals and multiculturalism in different cultural contexts (Knein et al., 2020) is important to study issues such as individual responses to crisis events in multicultural contexts.

Multiculturalism in all its forms has now become a major vehicle for globalization and modern values, which is an important arena for debate on the topic of national and religious identity (Hulewat, 1996). While multiculturalism tends to exist only symbolically in Europe, Japan, and other societies where modern values prevail, in developing regions, especially the Middle East, where traditional values and identities dominate the society multiculturalism is an exploration of the composition and regular expression of value systems. With the rapid development of society, the conflict of compartmentalization caused by intercultural communication is becoming more pronounced, which is ultimately not so much a clash between civilizations as a clash of cultural identity differences within civilizations (Lieber and Weisberg, 2002). Academics have generally found that, in addition to the obvious rules, specific mental and emotional empathic behaviors are altered by cultural context as well. Therefore, paying attention to individual psychological changes and coping strategies resulting from cultural differences based on different cultural environments enable us to gain a deeper understanding of why people behave accordingly in multicultural environments and how the results emerge as a function of cultural orientations and values (Main and Kho, 2020).

Based on the research focusing on the psychological and emotional characteristics of individuals in multicultural contexts, previous scholars have also conducted corresponding research on conflicts arising from the heterogeneous characteristics of cultural values (e.g., Han et al., 2021). Scholars have found that national cultural differences are potential factors stimulating the occurrence of crisis events, that cultural differences between countries are a major cause of conflict in international crisis events, and that the pattern of social crisis events caused by multicultural differences is universally applicable on a global scale (Yu et al., 2020). At the macro level, previous studies focused on cross-cultural conflict, ethnic stereotypes (Brigham, 1971), geographic distance (Li and Katsumata, 2020), and differences in cultural values (Cramton and Hinds, 2014). However, with the rapidly and dynamically changing society, the traditional conflict tracing model is hard to use to interpret the increasingly diversified cultural forms and the ever more enriched cultural dimensions. Therefore, it is necessary to further research the cultural differences and root causes of conflicts in transnational countries to explore the mechanisms by which cultural differences affect individual psychology, emotions, and behaviors in crisis events. This can explain the problems associated with social events in an era of frequent crises. However, research and references in this area still need to be enriched, and it is especially necessary to study the relationship and interaction between cultural differences and crisis event risk prevention and control in diverse countries. Therefore, we aims to explore the differences between global multicultural differences and their ways of preventing public crises and aim to improve the existing theoretical framework of cultural heterogeneity. This study aims to enrich the literature to deconstruct the connection between citizens' psychology, emotions, and active prevention mechanisms when facing social and international crises. Therefore, we propose:

***H1***: *Multicultural differences positively influence the occurrence of risk prevention and control behaviors*.

### The Meaning and Application of Empathic Communication in Crises

The application of empathy in the field of communication is currently a hot topic. Although the concept of empathy communication has attracted more and more attention by scholars in the study of public opinion research, unified definition is not clear yet. The current research on empathy communication mostly focused on the process of evaluating the effectiveness of communication, emphasizing the perceptual connection and similarity of the audience's perspectives. Broadly speaking, the emerging communication concept of empathy communication is derived from the psychological concept of empathy. Empathy was first translated from German by the British psychologist Edward Titchener in 1909 and means “feeling into” (Chen, 2017).

Since empathy was transferred to the field of psychotherapy by design aesthetics, it has long been a key concept of clinical psychology theory and practice. Thereafter, Carl Rogers systematically explained the psychological, emotional, and behavioral characteristics of applying the concept of empathy to cognitive psychotherapy (Rogers, 1958). According to Rogers (1958), the so-called empathy is the ability of one person to understand the unique experience of another and to react to it. This concept allows one person to have a certain amount of empathy for another person and then take action (Hyland-Wood et al., 2021). The view accepted by most scholars is that empathy can distinguish emotional cognitive components. In particular, the emotional component of empathy refers to an individual's response to another person's feelings, which is expressed as the consistency of one person's feelings with another person's emotional state (Cao et al., 2021; Zhou et al., 2021).

Up to date, studies have wildly applied the emotional and cognitive manifestations of empathy to the description of the thematic characteristics of communication audiences, management objects, and landscape visitors. As in contemporary social crisis events, empathy communication has a significant impact on events and social opinions, profoundly expressing the psychological, emotional, and behavioral feedback of the communication audience under the influence of the crisis. In the case of the COVID-19 outbreak in early 2020, information dissemination began to play an important role, as some countries recommended self-isolation to control the epidemic, while others recommended a conservative response measure to ensure normal life (Chen et al., 2020). The intervention of the media accelerated the spread of epidemic news and caused psychological fluctuations and emotional resonance effects in the information audience in the process of dissemination. Due to the spread of information about the COVID-19 epidemic, sympathy and preventive behaviors for the affected people began to spread in varying degrees over various regions. This is the product of the panic caused by the epidemic on media platforms. This panic has shaken the original normal social operating mechanism, affecting medical and health care, social security, and national stability (An et al., 2020). Thus, it is important to understand the transmitting process of empathy in a crisis, which is a key link in predicting the process of social development and an important guide for coping with group panic triggered by individual factors in crises. In the research related to the spread of empathy during the COVID-19 pandemic, some scholars correspondingly proposed the concept of a psychological typhoon eye for the description of public opinion distribution (Raza et al., 2020). This concept describes the irrational panic psychology and behavior of the public in major emergencies and provides a new idea for the study of an empathy communication mode, that is, the closer the public is to high-risk locations spatially, the calmer the public; the farther the public is from high-risk locations, the more they panic. The proposed communication mode is different from the original way of thinking about empathy communication, breaking the basic perception that the original analysts and participants of a crisis event must have a common feeling. The empathy-altruism hypothesis indicates that disasters often severely damage individuals' mental health (Cialdini et al., 1997), which is why providing positive, timely, and adequate psychological crisis intervention can help disaster victims, their families, and ordinary people overcome difficulties. Overall, the psychological impact of crisis events on people shows more negative effects at the root. How to better understand the audience's empathy caused by crisis transmission and to jointly explore ways and contradictions from time and perspective are both important. The spatial dimensions are a topic worthy of in-depth discussion. Therefore, we propose:

***H2:*** *Multicultural differences positively influence public empathy*.

### Risk Prevention and Control Within Multicultural Differences

Gallagher (1952) put forward the concept of risk management, which refers to the decision-making process of social organizations or individuals to reduce the negative results of risk. Risk management is a public organizational method that selects, optimizes, and combines various risk management techniques to effectively control risks, solve the losses caused by risks, and obtain maximum safety (Kraman and Hamm, 1999). The basic goal of risk management is to obtain maximum security at minimum cost, and risk prevention and control is an important part of risk management. At present, preventing and controlling the occurrence of crisis events is the main means to maintain social stability and social security. Due to the unpredictability of crisis events, most studies have idented that risk prevention and control of crisis events should be changed from passive to active. Specifically, active risk prevention and control exist not only at the macro level of policy governance, but also at the micro level which is equally important for individual active risk prevention and control. There is limited literature on individual active risk prevention and control. Compared with the social conflicts caused by multicultural differences, as well as the application of empathy communication in crises, it is necessary to focus on active prevention and control of individuals, and then formulate reasonable plans. In the modernization process of human society, and with the continuous development of technology and the economy, globalization and informatization have become more and more intense, and various social risks have frequently appeared, and even suddenly appeared in front of the public (Vicentini and Galanti, 2021). There is no doubt that after the outbreak of COVID-19, the world is at an important moment of public management. Governance systems have formed amid social concerns, and countries have introduced new polices one after another to actively prevent and control risks. How will governments, citizens, businesses, non-profit organizations, and the voluntary sector respond to a series of future large-scale social crisis events that will occur in the future? Can they be better prepared, react faster, coordinate better, and work more effectively, which is also the research focus of today's society (Lindquist, 2020). In the context of multinational cultures and empathy communication mechanisms, it is essential to make necessary changes and improvements to the existing macro-societal proactive prevention and control mechanisms or the ones being tested. At the micro level, a public crisis may be regarded as an individual's amorphous existence, but it can affect an almost unlimited number of individuals, groups, and organizations in the environment. Effective crisis management in a specific environment requires that individual citizens fully understand the public crisis. A large number of studies related to public crises have explored relevant laws, but there is almost no consensus on how to define the term (Borodzicz and van Haperen, 2002). The outbreak of the COVID-19 pandemic represents the obvious differentiation of individual active risk prevention and control in many public crisis events, and it is affected by various factors such as geography, culture, and thinking patterns. Therefore, to better understand the characteristics and laws of individual active risk prevention and control in public crises, it is necessary to explore both the differentiated characteristics and the common characteristics.

However, the roots of the differentiation and commonality of active risk prevention and control have not yet been revealed. In many contemporary crisis prevention and control cases, the impact of cultural differences has gradually attracted the attention of scholars, and the concept of cultural identity is considered as the cause of the characteristics of active risk prevention and control behavior. In the context of economic globalization, cultural identity has a certain impact on the stability of international communication (Friedman, 1994). Cultural identity is the commonsense cognition belonging to a certain group, and it is an important component of one's self-concept and self-cognition (Assmann and Czaplicka, 1995). It is reflected in race, religion, social class, generation, region, and any social ownership of a group with its own unique culture. Therefore, cultural identity is a characteristic of both individuals and groups with the same cultural background. At present, the academia believes that cultural identity includes three identities: personal identity, relational identity, and public identity (Jonker et al., 2020).

Cultural identity is an affirmative cultural value judgment, which refers to the recognition of the value utility of the emerging culture within the community or the foreign cultures outside the community by the members of the cultural group. It is an attitude and recognition method that conforms to traditional cultural value standards (Jonker et al., 2020). Huntington (1996) believes that cultural identity, which is an important basis for national identity and state identity, is the most important soft power in the overall performance of national power and has gradually become an important factor in dealing with crises. Cultural exchanges among countries can strengthen cultural identity, but they can also trigger conflicts in the collision. Although scholars have researched cultural identity, the current cultural identity concept has emerged along with modernity and the public crisis caused by it, and its new era characteristics have begun to gradually manifest (Zajda and Majhanovich, 2021). In terms of behavioral characteristics, there are highly significant differences in the ways in which different countries and people respond to the manifestations of crises. In the case of COVID-19, the government and the people in the Eastern cultural system paid more attention to epidemic prevention and control. However, in the Western cultural system, the government and the people advocated for liberalism and opposed restraints on life due to prevention and control. From a cultural perspective, both approaches are deeply rooted in cultural identity, and neither approach can be judged as right or wrong (Gato et al., 2021). Therefore, it is difficult to identify cultural differences between countries related to active risk prevention and control, and how to efficiently handle crises should be adapted to local conditions and form effective prevention and control behaviors that reflect a sense of cultural identity. Analyzing the similarities and differences between the characteristics of this multicultural empathy communication and the active prevention and control mechanism and exploring the regularity of multinational cultural identity in the resolution of crisis is a valuable guide for mankind when dealing with major crises in the future.

Multicultural heterogeneity determines significant differences in the response and handling of crisis events in local society. In a specific public crisis, empathy communication affects individual psychology and active risk prevention and control behavior. Although exist obvious differences among diverse cultures, the fact is undeniable that some common patterns are real. Researching and exploring the internal relationship between the common laws of different cultures and empathy communication is of positive significance for preventing and solving social crisis events and reducing the negative social impacts brought by transference of empathy. We aim to investigate the internal influence of cultural differences on empathy communication in a multicultural context, the relationship between these influences and the psychological and emotional reactions of individuals in society under crisis events, and the connection between proactive risk prevention and control behavior. It provides a more scientific action guide and theoretical reference for addressing public crises prevention and control under the background of multiculturalism.

In summary, multicultural differences lead to frequent social conflicts, while deviations in management concepts at the national and state levels make social laws appear incongruous. Management has begun to pay attention to the changes in public psychology and on the effect of empathy communication of crisis information to control those active risk prevention and control behaviors in the multicultural context. This study can tentatively conclude that the regulation of empathic psychology may become a key element of today's crisis management. Therefore, we propose:

***H3:*** *Empathy positively influences the occurrence of risk prevention and control behaviors*.
***H4:*** *The interaction between empathy and multicultural differences influences risk prevention and control behaviors*.

## Research Design

To further integrate theory with practice, this study selects COVID-19 as the research subject. And we are motivated to clarity the inherent laws of risk prevention and control guidance under the background of cultural differences through questionnaire surveys on the respondents whose differences in culture, psychology, and behavior of each country are apparent.

### Research Hypothesis and Theoretical Model

**Figure 1:**
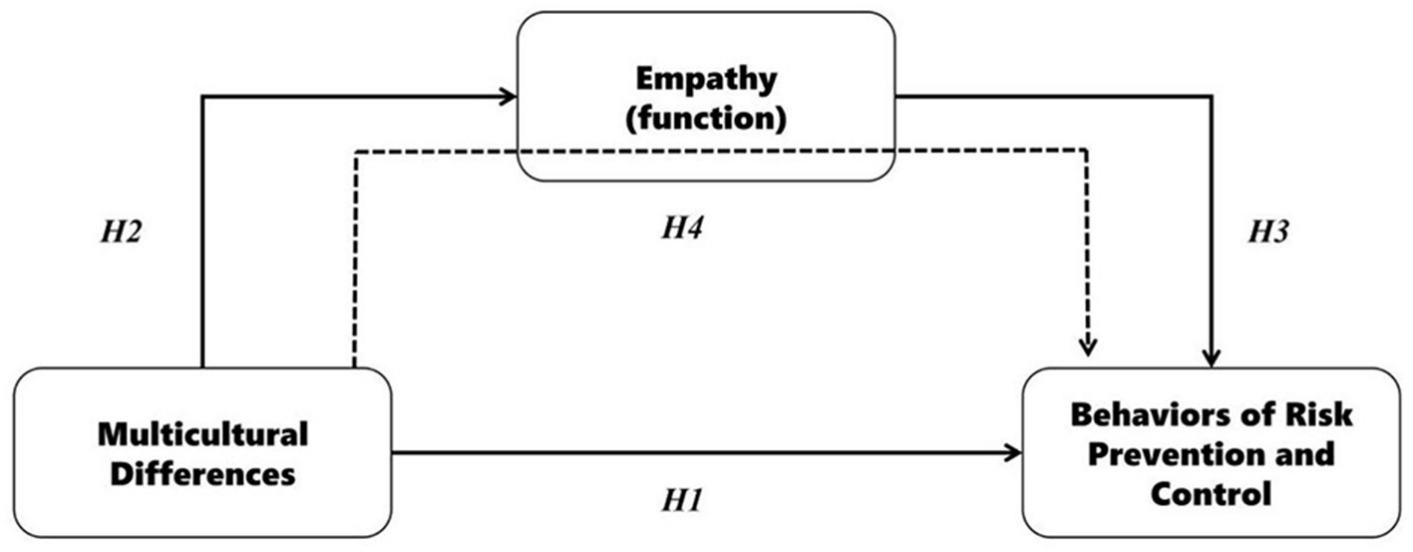
Research hypothesis model.

Based on the literature review and the understanding of this research question, it can be preliminarily drawn that the research on the behavioral guidance of risk prevention and control under the background of cultural differences focuses on the three levels of multicultural differences, empathy, and risk. This study should address four basics assumptions, and the theoretical framework is shown in Figure 1.

### Factor Characteristics and Question Design

To statistically measure the three factors of multicultural differences between empathy and risk prevention and control behaviors, this study summarizes the characteristics of these factors and the established scales.

#### Multicultural Differences

Since each country has its unique background of geography, humanities and history, and cultural integration, there are significant differences in humanities between countries, collectively referred to as cultural differences. Specifically, cultural differences refer to cultural differences unique to people in each country/region due to regional differences (Hofstede, 1980). In today's globalization of extensive exchanges and interactions, the concept of pluralism has begun to incorporate the idea of cultural differences. The academia has reached a consensus in a handful of research and explorations. Multiculturalism refers to the co-existence of multiple interrelated cultures. In a particular region, territory, society, group, or class system, everyone has independent cultural characteristics (Giardini and Wittek, 2019; Xu et al., 2020). It is different from the previous assumptions on cultural existence because it is diverse in space and extensive in time (Parekh, 2001). Academia generally believes that understanding and application of multiculturalism involves scientifically analyzing and exploring differences in space and time.

Academic circles have explored the factors that influence the differences of multiculturalism to measure their effectiveness in human society under realistic conditions (Sekiguchi, 2006; Li and Katsumata, 2020; Li et al., 2020, 2021). This study focuses on the impact of multicultural differences on public psychology and risk prevention and control behavior. Therefore, the Normative Multicultural Scale (NMS) is selected to measure the effects of multicultural differences on individuals in terms of policies and practices (Stuart and Ward, 2018). The scale enables us to systematically analyze the direct impacts of multicultural differences on interpersonal and intergroup communications at the level of multicultural ideology and multicultural policy, which has scientific significance.

#### Empathy

The classic concept of empathy has been applied in several fields, such as philosophy, sociology, and psychology (Eisenberg and Strayer, 1987). Although the formulation and application of the theory may seem ancient, it is still one of the main concerns of researchers (Bovina, 2020).

For the measurement of empathy, there are six measurement scales with high reliability: Negative Emotional Nature (NE) (Watson and Clark, 1984), Empathy Quotient (EQ) (Baron-Cohen et al., 2004), Basic Empathy Scale (BES) (Jolliffe and Farrington, 2006), Questionnaire for Cognitive and Affective Empathy (QCAE) (Reniers et al., 2011), Interpersonal Reactivity Index (IRI) (Davis, 1983), and Content Area of Extended Empathy (ACME) (Vachon and Lynam, 2015). Interpersonal Reactivity Index (IRI) and the Content Area of Extended Empathy (ACME) have been most adapted in prior studies. Previous literature reported that the ACME has more measurement breadth than the IRI and more comprehensively focuses on both positive and negative cognition of the empathic phenomenon (e.g., Murphy et al., 2020). Therefore, to better understand the role of empathy in cross-cultural communication, we adapted the ACME scale in this study. We conducted a questionnaire-based public psychological survey to investigate the rules of guiding risk prevention and control behaviors in the context of multi-culture at three levels, cognitive empathy, affective resonance, and affective dissonance.

#### Risk Prevention and Control

Risk prevention and control were first applied to the economic field and are proactive preventive behaviors chosen by business subjects in response to changes in the market or stock market. This preventive behavior can reduce economic losses and maximize financial outcomes. Later, crisis prevention and control were applied to various fields to observe the reaction of individuals in certain events. The effects of economic crisis on employee job satisfaction, commitment, and self-regulation scale are classic scales for measuring individual behaviors of risk control during crises (Markovits et al., 2014). Risk prevention and control behaviors also refer to social support in the field of psychology (Elklit et al., 2001). Social support is a multidimensional concept that includes both intra-individual cognitive and extrinsic environmental factors. In previous studies, researchers have developed a variety of measurement instruments from different perspectives, according to their different understandings of social support. The measurement of social support can be divided into two categories based on the definition of social support. One measurement is the content characteristics of social support and the other is the structural characteristics of social support. The concept of social support and coping style has been widely adapted and extended since the 1970s. The main scales on coping styles are the Ways of Coping Questionnaire (WCQ) scale developed by Folkman and Lazarus (1980), the COPE scale developed by Carver et al. (1989), and the Crisis Information Seeking and Sharing (CISS) scale developed by Lee and Jin (2019). Among those theories, the WCQ scale developed by Folkman and Lazarus (1980) has been widely applied in the studies of risk management (e.g., Jonker et al., 2020). In this study, individuals' behavior is mainly expressed in several dimensions of the reference scales CISS and WCQ, namely, individual autonomous behavior, socially active defense mechanism and security satisfaction, and affective commitment, therefore, the CISS and WCQ scales were adapted to this study.

### Sample and Method Selection

For data collection, people from different cultures in different countries under the influence of the COVID-19 pandemic were selected for the survey. To better explore the generalization patterns, a normal distribution of the sample was required due to the different levels of economic development in the study-restricted areas. To ensure that the selected sample qualified, the basic needs of the subject study were determined through selection. After obtaining a reasonable screening sample, an undifferentiated questionnaire survey was conducted on 320 subject who were significantly culturally heterogeneous and influenced by the role of empathy in crisis. The age of respondents covered 30–60 years old, and those who have formed mature and stable values.

This study applied questionnaires design, descriptive statistical analysis, and PLS-SEM to explore the logical relationship among multicultural differences, empathy, risk prevention, and control. First, the explanatory variables were screened by using a questionnaire, exploratory factor analysis, and descriptive statistical analysis. Second, the theoretical model was analyzed by using Smart PLS 3.3.3 software for confidence and validity tests. Third, we verified the four main paths. H1–H4 of the research hypotheses were verified by the influence coefficients of each path and the risk in the context of cultural differences was interpreted from the perspective of empathy. The final explanation of the mechanism of risk prevention and control behavior guidance in the context of cultural differences from the perspective of empathy was used to answer the three research questions in this study.

## Empirical Analysis

### Analysis of Demographic Variables

*To enrich the literature of risk prevention and control behaviors in the multicultural context*, the study constructed relevant questionnaires based on the existing scale index system (as shown in Table 1). Between May 2020 and January 2021, the team members distributed electronic questionnaires with the assistance of international cooperators. A total of 300 valid samples were obtained after screening and eliminating 20 from the 320 samples.

**Table 1:** Study scale and question items.

**Study scale**	**Items' tags**	**Measurement question items**
Multicultural differences (DW)	DW1	Your consciousness and values are different compared to people in other regions (value differences)
	DW2	Your information is delivered differently compared to people in other regions (habit differences)
	DW3	Your risk prevention and control measures are different compared to people in other regions (difference in needs)
	DW4	You understand things in a different direction compared to people in other regions (cognitive differences)
Empathic communication (GC)	GC1	You can distinguish the reasons why people act differently (discriminate)
	GC2	You can understand why people think this way (understanding)
	GC3	You can predict the actions of others (prediction)
	GC4	Helping people in need makes you feel good (feel)
	GC5	You don't care if people are happy or depressed (affective)
	GC6	When people are upset, you try to help them (subconsciously)
Risk prevention and control (FF)	FF1	You will change your habits when a crisis event occurs (change)
	FF2	You will promote your claims when a crisis event occurs (advocacy)
	FF3	You will tense yourself up when a crisis event occurs (heightened awareness)

The samples of this study were collected by survey at a college-level cultural industry management research center group. Therefore, this study was conducted relying on the relevant partner institutions of the host schools and colleges, as well as the personal partners of the research group members. The questionnaire was distributed online. The survey of this study was conducted in 10 countries, including China, Korea, Thailand, Japan, Pakistan, Russia, USA, Canada, Cameroon, and New Zealand, and 320 questionnaires were distributed. The survey process was divided into three main phases: the first phase was from May 2020 to September 2020; the second phase was from September 2020 to November 2020; and the third phase was between December 2020 and January 2021. A total of 20 questionnaires were excluded that lacked nationality information or if respondents finished the survey in less than 15 min, and finally 300 valid questionnaires were finally obtained, which provided the data basis for the subsequent empirical study (statistical characteristics are shown in Table 2). The preliminary data analysis of demographic variables reveals two main points. First, the study sample was distributed across all age groups who generally had a high level of education. Second, the sample had a broad geographical distribution and most of the participants had experienced sudden social crises. Therefore, the sample recovered was valid for further exploration.

**Table 2:** Statistical of demographic variables.

**Category**	**Demographic features**	**Quantity**	**Percentage**	**Category**	**Country name**	**Quantity**	**Percentage**
Gender	Male	152	50.67%	Region distribution	China	73	24.33
	Female	148	49.33%		Korea	32	10.67
Whether they suffered a sudden crisis	Yes	234	78.00%		Thailand	22	7.33
	No	66	22.00%		Japan	42	14.00
Age	Under 40	112	37.33%		Pakistan	23	7.67
	40–59	134	44.67%		Russia	23	7.67
	60 and over	54	18.00%		USA	19	6.33
Education	Non-bachelor	50	16.66%		Canada	23	7.67
	Bachelor	167	55.67%		New Zealand	28	9.33
	Post-graduate or above	83	27.67%		Cameroon	15	5.00

### Descriptive Statistical Analysis of Variables

The mean and standard deviation analysis of valid samples is the descriptive statistical analysis of the dimensions of multicultural differences, empathy, and behaviors of risk prevention and control. As required, the valid sample data for statistical analysis obeyed normal distribution, including the cultural difference variables DW1 (M = 4.33; SD = 0.801), DW2 (M = 4.21; SD = 0.762), DW3 (M = 4.30; SD = 0.819), DW4 (M = 4.15; SD = 0.834); the empathic psychological variables GX1 (M = 4.19; SD = 0.900), GX2 (M = 4.18; SD = 0.835), GX3 (M = 4.20; SD = 0.850), GX4 (M = 4.26; SD = 0.814), and GX5 (M = 4.34; SD = 0.752), GX6 (M = 4.26; SD = 0.772); and risk prevention and control behavior variables FF1 (M = 4.31; SD = 0.822), FF2 (M = 4.45; SD = 0.728), and FF3 (M = 4.33; SD = 0.785). All measurements were within the acceptable range of the measurement criteria, and thus the data were validated for the next statistical analysis.

### Reliability and Validity Analysis

For the reliability analysis, the focus was on the reliability, consistency, and stability of the measurement results, that is, whether the test results reflected the stable and consistent true characteristics of the test taker. This study then conducted an overall preliminary reliability test on the 13 valid data points recovered from the questionnaire scale: α = 0.943, indicating a good reliability.

For validity analysis, the effect of dimensionality reduction was achieved by extracting factors with eigenvalues >1. This study used orthogonal rotation to measure the intrinsic association between variables, simplifying the statistical data structure by reducing the dimensionality of the data. Deep analysis requires the judgement of data structure validity based on test statistic (KMO), Bartlett's test, contribution rates, and factor loadings. This study tested the validity of the data structure of the three dimensions proposed by the hypotheses. The results showed that the KMO value was 0.953, which was >0.8; the significance was 0.00, which was <0.05, indicating that the validity was good enough and the data qualified the need of structural equation model analysis.

On the other hand, to further verify the validity of the test sample under multicultural differences, the study measured the validity and reliability of the country-specific samples (as shown in Table 3). The Cronbach's alpha coefficient was greater than 0.9 in all countries, the KMO value was 0.948, and the Bartlett's test of sphericity approximation chi-square was 3297.955, indicating that the validity was good enough and the data qualified the need of structural equation model analysis (Bland and Altman, 1997).

**Table 3:** Validity and reliability tests of countries.

**Country name**	**Sample size**	**Cronbach's alpha**	**KMO and Bartlett's test**
China	73		
Korea	32	0.973	
Thailand	22	0.964	
Japan	42	0.957	
Pakistan	23	0.904	KMO = 0.948, and the Bartlett's test of sphericity approximation chi-square was 3297.955
Russia	23	0.965	
USA	19	0.977	
Canada	23	0.973	
Cameroon	15	0.909	
New Zealand	28	0.965	

### Structural Equation Modeling Based on Smart-PLS

This study applied the partial least squares method, which is suitable for small sample analysis and can handle non-normally distributed data. Since the PLS method can overcome the problem of covariance among observed variables and remove the effect of unhelpful noise on the regression, it can endow the PLS model with better robustness (Sarstedt et al., 2019). Therefore, PLS-SEM was selected as an effective tool for exploration in this study.

**Figure 2:**
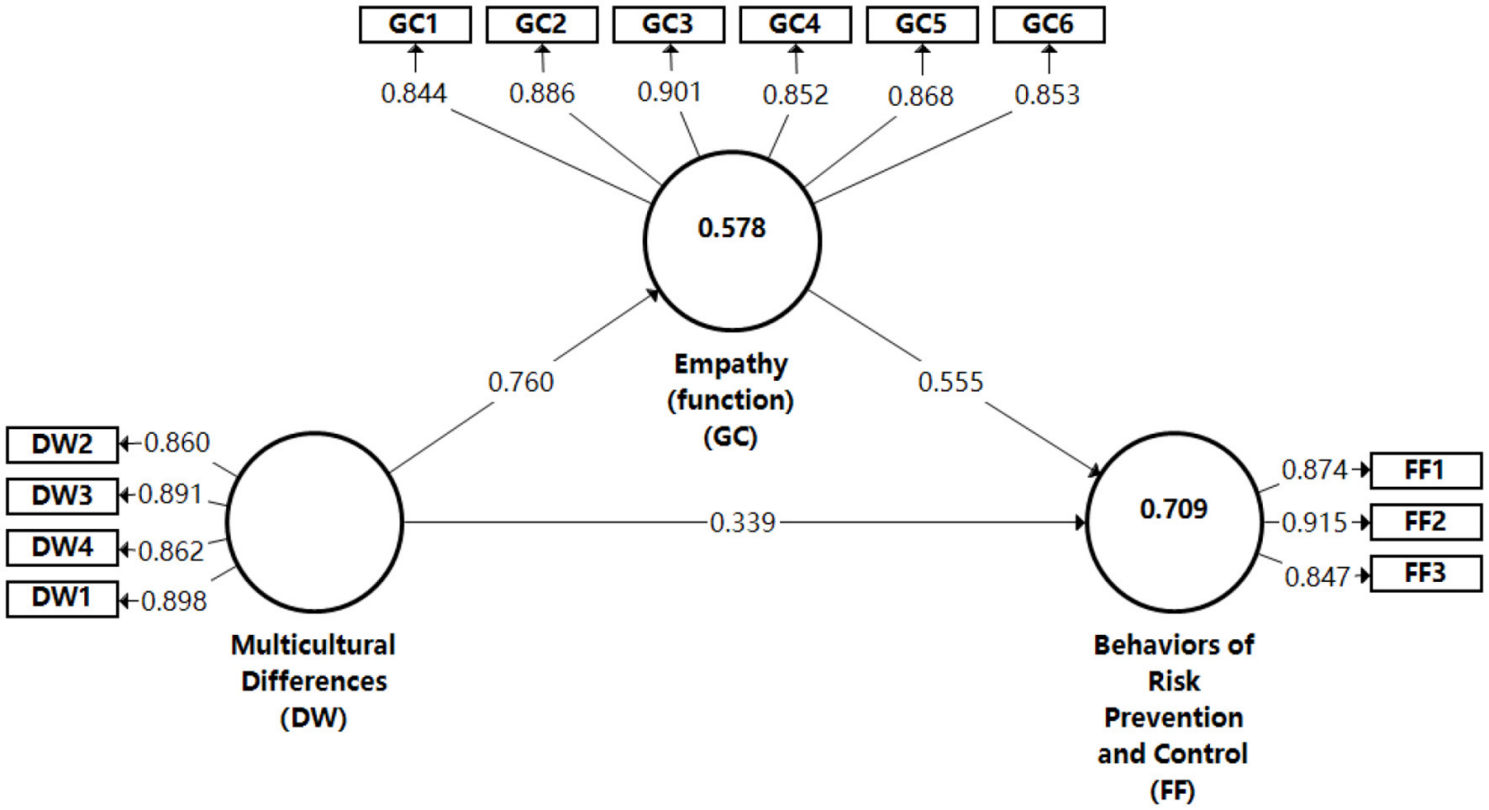
Reflective measurement model.

Specifically, a single dimensional test was conducted on the secondary indicators corresponding to the three primary indicators. The results showed that the eigenvalues of the first principal component of each dimension were >1, and the eigenvalues of the remaining principal components were <1. All dimensions passed this test. Then, using Smart PLS 3.3.3 software and the PLS algorithm, the intrinsic mechanism model of multiculturalism and empathy in crisis was established by the reflective measurement model (Figure 2), and the normative fit index (NFI) was 0.868 (>0.8), which reached the model fit requirements well.

The results of the model show that, except for the evaluation item of risk prevention and control, the other two latent variables represented two primary indicators, and the 13 secondary indicators were composed of dimensional item codes and serial numbers. The PLS algorithm is used for analysis, the software is standardized and used for the following analysis. The results show that the model fit well; and had significant explanatory utility for the internal potential relationships; the estimation effects were all acceptable; and the reliability indicators fit the structural validity, with the specific parameters shown in Table 4.

**Table 4:** Model reliability test results and fit indices.

**Indicators**	**Cronbach's alpha**	**rho_A**	**CR**	**AVE**	***R* ^2^**	**Q^2^**
Empathy (GX)	0.934	0.935	0.948	0.753	0.709	0.567
Multicultural differences (DW)	0.901	0.907	0.931	0.771		
Behaviors of risk prevention and control (FF)	0.852	0.856	0.911	0.773		

The *R*^2^ value of risk prevention and control in Table 4 is 0.709, which indicates that each latent variable had strong explanatory power for risk prevention and control. The Cronbach's alpha coefficients of each latent variable were all >0.7, indicating that each latent variable has good reliability. The combined reliability CR of each latent variable satisfies the requirement of >0.7, which proves the high reliability of the model. The average extracted variance AVE and rho_A of each latent variable were >0.5, and thus reached the relevant statistical criteria (Fornell and Larcker, 1981).

Further, we assessed the influences of Q^2^ exogenous variables on endogenous variables, and acquired a Q^2^ >0.35, which indicates a higher influence of the exogenous variables on the endogenous variables and indicates that the predictive relevance of the model is stronger. The value of Q^2^ in Table 2 is 0.567, indicating that the exogenous variables of this model have strong predictive relevance to the endogenous variable of the level of comprehensive development, indicating that the predictive power of the PLS model is stronger (Hai et al., 2019).

To test the correlation coefficients, a matrix of correlation coefficients among the latent variables was constructed, as shown in Table 5. The diagonal line is the open root sign value of the average extracted variance (AVE) of each latent variable. The values below the diagonal value are the correlation coefficients among the latent variables, respectively. Comparing these two sets of correlation coefficients indicates that the latent variables have different theoretical connotations and have good differential validity.

**Table 5:** Correlation coefficient matrix between latent variables.

	**Empathy (GX)**	**Multicultural differences (DW)**	**Behaviors of risk prevention and control (FF)**
Empathy (GX)	0.868		
Multicultural differences (DW)	0.760	0.878	
Behaviors of risk prevention and control (FF)	0.813	0.761	0.879

The t-statistic of each path coefficient was calculated using Bootstrapping; the significance level of the path coefficient estimates was tested (two-tailed test) as shown in Table 6. If 2.58 > T > 1.96 then the path coefficient estimates were significant at the 0.05 level. If 3.29 > T > 2.58 then the path coefficient estimates were significant at the 0.01 level. In the Bootstrapping test, all path coefficients had high t-statistics of the structural equation model. All path coefficient passed the test at the corresponding significance level and the stability of the model structure was good (Streukens and Leroi-Werelds, 2016).

**Table 6:** Significance test results of path coefficients.

	**O**	**M**	**STDEV**	**T**	***P***
Empathy (GX) → Behaviors of risk prevention and control (FF)	0.555	0.550	0.070	7.976	0.000
Multicultural differences (DW) → Empathy (GX)	0.760	0.764	0.049	15.419	0.000
Multicultural differences (DW) → Behaviors of risk prevention and control (FF)	0.339	0.346	0.066	5.106	0.000

To verify whether multicultural differences would impact individual risk prevention and control through empathy, the study used the Bootstrapping method to calculate the coefficient of the specific mediated path. The results showed that the mediated path had a reliable mediated performance, with the specific parameters shown in Table 7. The study finally obtained the PLS-SEM. The following interpretations were made based on the path coefficients of the structural equation model and the previous theoretical derivations. The model fitting test available to estimate whether multicultural differences positively influenced the occurrence of risk prevention and control behaviors (H1). When social crisis events occur, different cultural groups take different measures of prevention and control behavior in the process of coping, which generally stems from the direct influence of values. In the process of risk prevention and control, the influence of diverse cultural differences on the individual behavior of risk prevention and control is not significant. Because the cultural identity carried by individuals in the macro environment of multicultural society cannot directly influence the decision making of the society, and the cultural identity of individuals may even be weakened by other factors.

**Table 7:** Results of the specific mediated path test.

**Specific mediated path**	**O**	**M**	**STDEV**	**T**	***P***
DW → GX → FF	0.422	0.419	0.050	8.427	0.000

Heterogeneous manifestations of multiculturalism drive different changes in empathic psychology, thus positively influencing the changes in public empathy, proposed in H2. In a multicultural context, empathy is often influenced by the efficiency and content of cultural communication, thus it varies with the way different cultures receive and process information. In short, the cognitive level and reception patterns of social individuals in different cultural systems are influenced by their native environment and have certain limitations. The real social environment indirectly leads to differential understanding and omission of information transmission between the two cultural groups in the process of emotional transmission, and the differences will be reflected in the fluctuation of emotions of different cultural groups in response to social crises.

The impact of empathy on risk prevention and control behavior is intuitively visible, and H3 is both a test of previous perceptions and a secondary validation based on the data. The emergence of new media has accelerated the dissemination of crisis information. The emotional activity of individuals influenced by media information is the ideological basis of practical behavior thus the emotional impact of crisis information largely determines the strength of their risk prevention and control. For example, the mass panic effect of the COVID-19 pandemic does not essentially come from the crisis itself, but rather from the direct impact of the spread of empathy, and the spread of panic causing the emergence of overreaction as a reflection of this principle.

Combined with the above model, we conclude that multicultural differences are an important consequence for the differentiation of individual behaviors of risk prevention and control in crisis events. The strength of risk prevention and control behaviors is influenced by the combination of information accuracy and completeness in the process of empathy generation. Differences in culture and cognitive structure are important consequences for the lack of information accuracy and completeness, which can verify the value of the psychological mediating utility of empathy in H4. It demonstrated that empathic psychology as a mediating variable in the context of multicultural differences can interact together with empathy, which can impact public risk prevention and control behaviors and their differential performance in crisis events.

Furthermore, this study verifies the mediating role of empathy in risk prevention and control in a multicultural context, and further concentrate the research on how culture drives behavior. Verifying the cognitive and emotional empathy characteristics can be interpreted through the correlation coefficients of specific question items, which both modulate risk prevention and control behaviors. Therefore, the public behaves differently when faced with COVID-19 in each country. Based on those points, the study also highlights that indirectly regulating the public's perceptions and emotions is a better option than directly issuing policies against crises.

## Further Exploration of the Logic of Risk Prevention and Control Behaviors in the UGC Era

### Background of the UGC Era and the Actor of Risk Prevention and Control

User-generated content (UGC), and its core embodiment, means that each individual can be both a communicator and an audience. Since social networks have become a key source of information acquirement, cognition, emotion, and behavior have changed to accommodate a new mode of expression.

Based on the previous research, there is a typical cause and effect logic in information dissemination, and the actors of risk prevention and control are the disseminators and audiences of information. Information is disseminated in very diverse channels in the UGC era, the disseminators and audiences blur the distinction and show cyclical turnover.

### Occurrence of Risk Prevention and Control Behavior in the Context of UGC

Periodicity is the internal logic of the identity of actors changing risk prevention and control in the UGC era and is more detailed under the mechanism of culture, empathy, and risk prevention and control. Specifically, the first disseminator of crisis information can be either the official media or the public, and the former has a certain authority, while the latter highlights the universal characteristics of content dissemination. In this era, communicators tend to be more subjective and more inclined to share their own feelings and potential perceptions, and thus the background of the UGC strengthens the motivation of communicators in the early stage of information dissemination. Therefore, communicators begin to enter the culture, empathy, and risk prevention and control mechanism, meanwhile multicultural differences strongly influence the risk prevention and control behavior, highlighting the mediating role of empathy psychology, which in turn shows that risk prevention and control behavior is dominated by emotion. Behavior originates from the audience and then influences others, the group effect is the significant factor currently. The background of the UGC era again presents its promotional effect, the influenced audiences are transformed into the information disseminators.

### Circular Mechanism of Risk Prevention and Control in the Context of UGC

The first occurrence of risk prevention and control behavior brings about the first shift in audiences' identity. Existing perceptions, emotions, and behaviors are the basis for the new communicators to process information (Luqman et al., 2021; Sun, 2021; Sun et al., 2021). The information at the beginning of the communication has its original character and would be accompanied by the rational analysis of the actors in the second round of communication, and the social imprint of multiculturalism has been burdened from then on. The background of UGC again stimulates communicators to initiate secondary information dissemination. Compared with the official media, the secondary communication has become more prominent. Information transmission is more clear and rapid under the influence of the culture, empathy, and risk prevention and control mechanism. The risk prevention and control behavior of the new audience again generates a group effect. With the promotion of the UGC, the audience has a certain chance to become new communicators, so the third round of crisis information dissemination begins.

Communicators and audiences gradually stabilize after rounds of risk prevention and control behaviors together with the mechanism of culture, empathy, and risk prevention and control. Due to the decoding consciousness of each actor of risk prevention and control behavior, the irrational component of information has been reduced in the information flow, as well as the continuous influence of multicultural differences, which further demonstrates the centrality of empathy in the process of risk prevention and control behavior. The audience in each round of information dissemination expresses their demand for information feedback, which gives rise to the anti-driving mechanism for information disseminators. The logic of risk prevention and control behavior in the UGC era is becoming clearer.

## Conclusion and Recommendation

Due to the frequent occurrence of social crises, how to properly deal with social crises and minimize the losses caused has become a hot topic. In the experience of fighting COVID-19, excessively positive or negative risk prevention and control measures have negative impacts on the healthy development of society and the economy. Exploring the relationship of culture and empathy prevention and control is useful for handling the social crisis in the long run. This study explores the internal influence mechanism among multicultural differences, empathy communication performance, and individual risk prevention and control behaviors in crisis events from the theoretical evolution level. This study reveals the internal laws of multicultural differences through the intermediary variables of empathy communication and empathy. They have jointly regulated the differentiated performance and intensity of public crisis risk prevention and control behavior. This study further discusses the occurrence logic of risk prevention and control behaviors in the context of the UGC era. It also sorts out the actors, the first occurrence characteristics, and the cyclical mechanism of occurred behaviors. This study clarifies the value of empathic psychology as a code in the present time and reacts to its cyclic characteristics.

This study can answer the three initial questions raised by the survey results. First, as shown by empirical analysis, the coefficient of multicultural differences behaviors of risk prevention and control shows that multicultural differences positively impact risk prevention and control behaviors, verifying that cultural differences are one of the influencing factors of risk prevention and control behaviors. Reasons for different risk prevention and control behaviors explain, in the current COVID-19 epidemic, why different countries have different risk prevention and control behaviors. Second, the empirical analysis uncovered the coefficient of empathy behaviors of risk prevention and control, which indicates that empathy has a greater impact on individual behavior. Third, the coefficient of multicultural differences, empathy, and the correlation prove that empathy is a direct factor influencing the occurrence of risk prevention and control behavior.

Based on these findings, this study puts forward the following three suggestions for the guidance of social crisis risk prevention and control behavior. First, from the perspective of the public, information audiences should analyze multicultural characteristics of the society they live in, select information sources, objectively analyze the severity of the crisis events in the multicultural context, and apply the basic logic of cultural heterogeneity and empathy in their life practices. Internet information often affects the emotions and behavioral logic of individuals in society, resulting in many excessive prevention and control practices in social crises. Individuals should carry out reasonable crisis prevention and control practices according to their own needs. Second, the perspective of social public media and personal self-media should pay attention to the accuracy and objectivity of information dissemination, tolerate5 misunderstanding of multiculturalism, give due explanations to differences in understanding, and avoid culture as much as possible. The integration of cultural differences and emotional factors indicate that the news media that reduces negative social impact becomes the fuse that ignites social crisis. Social media is one of the social responsibility bearers and should not pursue economic benefits to the neglect of social public interests. Third, from the perspective of the government, government power should function appropriately in social crises. The government is a servant of the public, not a creator of cultural conflicts. Considering the multicultural differences in the management field, regulating the negative spread of excessive emotions in crisis events, reducing the cultural and emotional conflicts triggered by crises, and using the internal operation of culture and empathy to regulate social influence should be the focus of local governments.

Our study effectively explored the behavior-oriented risk prevention and control mechanism in a multicultural context, but there are still some aspects to be improved. First, the main source in this paper are the data of first-hand interviews, which are subject to geographic restrictions. Although the research team has collected data from 10 different countries, the accuracy of the data still needs to be improved, and we will continue the research in the future to analyze the behavioral patterns hidden under cultural differences by obtaining data covering more countries. Second, this study explains the psychological mechanism of behavior from the perspective of empathy, however, the emergence of behavior is not only limited by people's mental activities, other influencing factors such as social environment, group interaction, and economic context also deserve further study. Third, we conducted empirical analysis to verify the logic of risk prevention and control in the contemporary multicultural context. As time passes and the world environment changes, the pattern is bound to change, and comparative analysis will be conducted for multiple time dimensions in the future to enhance the research value of the findings.

## Data Availability Statement

The raw data supporting the conclusions of this article will be made available by the authors, without undue reservation.

## Ethics Statement

The studies involving human participants were reviewed and approved by East China Jiaotong University. The patients/participants provided their written informed consent to participate in this study.

## Author Contributions

KY contributed to the empirical work, to the analysis of the results, and to the writing of the first draft. YL and XW supported the total work of the first author. HP contributed to overall quality, and supervision the literature organization. RT contributed to developing research hypotheses and revised the overall manuscript. Authors discussed the results and commented on the manuscript.

## Funding

This project was supported by National Social Science Foundation, Nos. 15BXW052 and 19BXW097; Social Science Planning General Project in Jiangxi Province, No. 21XW06; Jiangxi Province Culture and Art Science planning general project, No. YG2021087; Jiangxi Province Colleges Humanities and Social Science Project, No. GL20214; National Natural Science Foundation No. 71772129; and General project of Humanities and Social Science Planning Foundation of Ministry of Education, No. 19YJA810002.

## Conflict of Interest

The authors declare that the research was conducted in the absence of any commercial or financial relationships that could be construed as a potential conflict of interest.

## Publisher's Note

All claims expressed in this article are solely those of the authors and do not necessarily represent those of their affiliated organizations, or those of the publisher, the editors and the reviewers. Any product that may be evaluated in this article, or claim that may be made by its manufacturer, is not guaranteed or endorsed by the publisher.
